# Radiation therapy alone versus radiation therapy plus radiofrequency ablation/vertebral augmentation for spine metastasis: study protocol for a randomized controlled trial

**DOI:** 10.1186/s13063-020-04895-x

**Published:** 2020-11-23

**Authors:** Rupesh Kotecha, Brian J. Schiro, Justin Sporrer, Muni Rubens, Haley R. Appel, Kathleen S. Calienes, Belinda Boulanger, Marietsy V. Pujol, Deborah T. Suarez, Ashley Pena, Alex Kudryashev, Minesh P. Mehta

**Affiliations:** 1 Department of Radiation Oncology, Miami Cancer Institute, Baptist Health South Florida, Office 1R203, Miami, FL 33176 USA; 2 Herbert Wertheim College of Medicine, Florida International University, Miami, FL USA; 3 Vascular and Interventional Radiology, Miami Cardiac and Vascular Institute, Baptist Health South Florida, Miami, FL USA; 4 Neuroscience Center, Baptist Health South Florida, Miami, FL USA; 5 Office of Clinical Research, Miami Cancer Institute, Baptist Health South Florida, Miami, FL USA

**Keywords:** Radiation therapy, Radiofrequency ablation, Vertebral augmentation, Spine, Vertebrae, Bone metastasis, Randomized controlled trial

## Abstract

**Background:**

Spine metastasis is a common occurrence in cancer patients and results in pain, neurologic deficits, decline in performance status, disability, inferior quality of life (QOL), and reduction in ability to receive cancer-directed therapies. Conventional external beam radiation therapy (EBRT) is associated with modest rates of pain relief, high rates of disease recurrence, low response rates for those with radioresistant histologies, and limited improvement in neurologic deficits. The addition of radiofrequency ablation/percutaneous vertebral augmentation (RFA/PVA) to index sites together with EBRT may improve pain response rates and corresponding quality of life.

**Methods/design:**

This is a single-center, prospective, randomized, controlled trial in patients with spine metastasis from T5-L5, stratified according to tumor type (radioresistant vs. radiosensitive) in which patients in each stratum will be randomized in a 2:1 ratio to either RFA/PVA and EBRT or EBRT alone. All patients will be treated with EBRT to a dose of 20–30 Gy in 5–10 fractions. The target parameters will be measured and recorded at the baseline clinic visit, and daily at home with collection of weekly measurements at 1, 2, and 3 weeks after treatment, and at 3, 6, 12, and 24 months following treatment with imaging and QOL assessments.

**Discussion:**

The primary objective of this randomized trial is to determine whether RFA/PVA in addition to EBRT improves pain control compared to palliative EBRT alone for patients with spine metastasis, defined as complete or partial pain relief (measured using the Numerical Rating Pain Scale [NRPS]) at 3 months. Secondary objectives include determining whether combined modality treatment improves the rapidity of pain response, duration of pain response, patient reported pain impact, health utility, and overall QOL.

**Trial registration:**

ClinicalTrials.gov NCT04375891. Registered on 5 May 2020.

## Background

Metastasis to the spine is a common complication of cancer, with approximately 40% of patients developing clinically significant disease. In autopsy series, up to 90% of patients with metastatic cancer have been identified as having micrometastatic spine disease [1]. Bone pain from spine metastasis is associated with increasing disability rates in these patients [2]. Furthermore, unlike other bone metastasis, spine metastasis results in vertebral body instability, sensory and motor neurologic deficits, and spinal cord or cauda equina compression, which can compromise a patient’s functional status and ability to receive other cancer-directed therapies. The standard treatment for patients with spine metastasis is fractionated external beam radiotherapy (EBRT), typically delivered in 1–10 fractions. The Radiation Therapy Oncology Group (RTOG) 97-14 trial randomized patients with bone metastasis (including spine disease) to 8 Gy in 1 fraction or 30 Gy in 10 fractions and demonstrated comparable outcomes between both arms, leading to the utilization of both schedules in clinical practice [3]. However, the duration and rate of pain control for the subset of patients with spine metastasis in both arms was quite modest, with only 61% of patients reporting complete or partial pain relief 1 month post-treatment. An alternative approach for patients with localized spine metastasis is spine stereotactic radiosurgery (SRS). Although retrospective series with this technique are promising, they remain unvalidated in the absence of prospective randomized data [4]. In addition, it is unclear how many patients are eligible for this highly precise treatment given that more than 50% of patients with spine metastasis have involvement across multiple levels and up to 38% have involvement of multiple, non-contiguous segments [5]. Further, there is a defined rate of late vertebral collapse consequential to SRS, which can worsen clinical symptoms.

Radiofrequency ablation, with or without percutaneous vertebral augmentation (RFA/PVA), has been used to treat pathologic fractures associated with spine metastasis with high rates of initial pain response [6]. Using this technique, disease in the central portion of the vertebral body, or that approaching the posterior elements, is first treated with lethal thermal radiofrequency (RF) energy, and the remaining bone is reinforced with cement to provide stability. Retrospective series have demonstrated significant early pain response and reduction in narcotic use as early as 1 week post-treatment [7]. Investigation into the benefits of a multi-modality approach is warranted, as RFA offers rapid pain relief but has limited oncologic effect by not addressing the entirety of the vertebral metastatic disease, especially in lesions in paraspinal locations and with disease in posterior elements. A small, non-randomized feasibility study of RFA and EBRT forms the basis of the combined modality approach investigated in this trial. In this study of 15 patients with bone metastasis and a pre-treatment pain score of at least 5 treated with RFA and EBRT (20 Gy in 5 fractions) matched to patients treated with EBRT alone, higher rates of complete pain response (53.3 vs. 16.6%, *p* = 0.027) and overall pain response (93.3 vs. 59.9%, *p* = 0.048) were observed [8]. This study was limited by the RF technology available at the time, lack of PVA in at-risk patients, as well as small sample size; therefore, further randomized prospective evidence is needed to characterize the benefits of modern combined modality techniques.

The primary objective of this randomized, controlled trial is to determine whether RFA/PVA in addition to EBRT improves pain control compared to EBRT alone for patients with spine metastasis. Secondary objectives include determining whether combined modality treatment improves the rapidity of pain response, duration of pain response, patient reported pain impact, health utility, and overall quality of life (QOL).

## Methods/design/interventions

This study is a single-center, prospective, randomized, controlled trial of patients with spine metastasis with two arms. The study protocol has been reported in accordance with the Standard Protocol Items: Recommendations for Clinical Interventional Trials (SPIRIT) guidelines (see Additional file 1). Eligible patients will have spine metastasis from T5 to L5 (within the treatment limits of RFA/PVA). There is no limit to the maximal involvement of contiguous vertebral bodies with metastatic disease, as long as an index lesion of pain can be determined and fewer than two sites would undergo RFA/PVA in total. All patients will undergo an MRI of the involved spine within 6 weeks prior to registration to determine the extent of spine involvement and must score their index lesion-associated pain as at least 5 using the Numerical Rating Pain Scale (NRPS). In the control arm, patients will receive conventional palliative EBRT to any of the following dose and fractionation schedules per the discretion of the treating physician: 20 Gy in 5 fractions, 24 Gy in 6 fractions, and 30 Gy in 10 fractions. Single fraction radiotherapy is not allowed in this study since this has been associated with higher incidence of re-treatment [9] and that fractionated regimens are more traditionally used in patients who have undergone percutaneous or surgical interventions. Patients randomized to the experimental arm will undergo RFA/PVA followed by EBRT using the same dose/fractionation schedules. All supportive therapy for optimal medical care will be given during the study period at the discretion of the attending physician(s) within the parameters of the protocol and documented as concomitant medications. Chemotherapy is not permitted within 24 h prior to or concurrently with EBRT. In addition, the patient can receive chemotherapy no earlier than 24 h after EBRT. If a deviation from the protocol occurs, the PI will complete and electronically submit the “Protocol Deviation Form.”

**Fig. 1:**
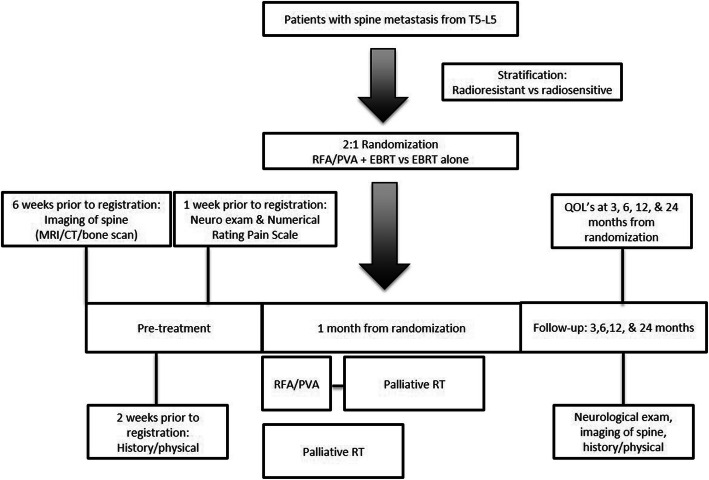
Flowchart of the clinical trial

The target parameters will be measured and recorded at the baseline clinic visit (on the day of treatment) and daily at home with collection of weekly measurements at 1, 2, and 3 weeks after treatment, and at 3, 6, 12, and 24 months following treatment with imaging and QOL assessments (see Table 1). There will be no special criteria for discontinuing or modifying allocated interventions given that both are standard of care interventions (Fig. 1).

**Table 1:** Study parameter table

Assessments	Pre-treatment	1 month from randomization	Follow-up: At 3, 6, 12, 24 months from randomization
History/physical	Within 2 weeks prior to registration		X
Performance status	Baseline		X
Neurological exam	Within 1 week prior to registration		X
Imaging of the spine (CT scan/bone scan is allowed, MRI preferred)	Within 6 weeks prior to registration		X
Numerical rating pain scale and documentation of patient’s pain medication^a^	Within 1 week prior to registration	(On the day of treatment) (At home: Daily with cumulative weekly measurements at 1, 2, and 3 weeks; bring to clinic at 1 month) In clinic at 1 month	X
FACT-G (FA), BPI (QL), EQ-5D (HP)	Baseline		At 3, 6, 12, and 24 months from randomization
Adverse event evaluation		X	X

^a^NRPS at 3 months is required for analysis of the primary endpoint

### Recruitment and randomization

All patients will be enrolled in this trial at the treating institution, Baptist Health South Florida, Inc. Patients will be provided with information about this study by the medical personnel and research staff in the radiation oncology department when evaluated in consultation for palliative EBRT to spine metastasis. The medical charts of interested patients will be reviewed by the research staff to ensure each patient meets eligibility criteria. Radiation oncologists will discuss patient eligibility for the trial with the interventional radiology and neurosurgical teams and review relevant imaging to determine the sites for RFA/PVA. Patients will also meet with the procedural team, either neurosurgery or interventional radiology, to discuss the logistics, risks, and benefits of the procedure. Given this patient population with metastatic disease and the follow-up intervals occurring at routine time points and coordinated with imaging studies, it is expected that the combination of these will help boost participant retention and completion of follow-up events. All patients who enroll onto this clinical trial will sign an informed consent document which outlines the research study in detail. The principal investigator or sub-investigators will describe the study to the patient and obtain written informed consent.

Patients enrolled onto this trial will be stratified according to the tumor type (radioresistant [soft tissue sarcoma, melanoma, and renal cell carcinoma] versus other non-hematologic tumor histologies). The treatment allocation scheme will be used for randomization to balances these patient factors [10]. Within each stratum, patients will be randomized in a 2:1 ratio to either RFA/PVA and EBRT or EBRT alone. This unequal randomization was chosen given the well-known and documented pain response rates to EBRT, accommodates increased demand for combined modality treatment, allows for a larger sample in the combined modality group to evaluate for potential adverse events, and follows the paradigm established by RTOG 0631. The randomization will be performed by a statistician using stratified permuted block randomization. Allocation concealment technique will be used to prevent selection bias and will be performed on an offsite computer. The recruitment phase will conclude when 52 patients have enrolled in the trial (35 in the RFA/PVA and EBRT arm and 17 in the EBRT alone arm), and it is expected that accrual should be completed over a course of 18 months.

### Inclusion criteria

The target population for this trial includes patients with metastatic disease to the spine, between T5 and L5, as detected by any imaging study. There is no limit on the maximal involvement of contiguous vertebral bodies of disease as long as the index lesion(s) can be treated with RFA/PVA and fewer than 2 sites need to undergo treatment in total. Patients must have a Zubrod performance status of 0–3 and can have other sites of visceral or osseous metastatic disease. Patients with epidural extension of disease are eligible for this study provided that there is ≥ 3-mm distance between the spinal cord and the edge of the metastasis. Key ineligibility criteria include metastatic disease from radiosensitive histologies (myeloma, lymphoma, small-cell lung cancer, or germ-cell tumors), those with frank spinal cord compression, or those with rapid neurologic decline or bony retropulsion necessitating urgent neurosurgical intervention.

### Assessment of the primary and secondary endpoints

The primary endpoint for this trial is pain response, defined as complete or partial pain relief, as measured using the NRPS at 3 months (post-treatment pain score of 0 or at least 3 NRPS points). The NRPS is a widely utilized assessment of pain intensity in oncologic clinics and has demonstrated reliability in studies of small sample sizes and in settings where repeated measurements are obtained to monitor changes in pain intensity over time [11]. Using this scale, complete pain relief is defined as a pain score of 0 with no increase in narcotic pain medication, and partial pain relief is defined as a reduction in numerical pain score of at least 2 points at the index site with no increase in pain medication.

Secondary pain response endpoints include the rapidity of pain response, as defined as the time from study entry to complete or partial pain relief, and duration of pain response, as defined as the time from complete or partial pain relief to pain worsening (≥ 3 NRPS points). To assess these outcomes, patients will complete the NRPS for each treatment site at the baseline clinic visit (on the day of treatment) and daily at home with weekly measurements at 1, 2, and 3 weeks after randomization in addition to a diary of their pain medication use and any side effects they experience related to the treatment. One month after treatment, patients will complete the NRPS, and at the clinic visit, the research team will consolidate the patients’ pain level scores, pain medications, and document adverse events. Imaging of the treated spine will be obtained at baseline and at 3, 6, 12, and 24 months after randomization to assess the response to treatment as well as subacute or long-term change of the vertebral bone.

The study hypothesis is that given the potential benefit of combined modality treatment in providing rapid and durable pain relief, QOL will improve after RFA/PVA and EBRT. Therefore, a number of QOL outcomes will also be prospectively collected in this study, including QOL, as measured by the Functional Assessment of Cancer Therapy (FACT-G) [12]; patient perception of pain, as measured by the Brief Pain Inventory (BPI) [13]; and health utilities, as measured by the EuroQol (EQ-5D) [14] at 3, 6, 12, and 24 months post-study randomization.

### Radiotherapy

All patients enrolled onto this clinical trial will receive EBRT (Fig. 1). CT simulation will be performed after randomization in a stable and comfortable position to allow for reproducibility from simulation to treatment. A variety of immobilization systems may be utilized, depending on the location and extent of spine disease to be treated. Image co-registration of the diagnostic MRI to the simulation CT can be performed if there is concern for soft tissue tumor component or epidural extension of disease. The gross tumor volume (GTV) contour represents the index spine lesion(s) and should include the entirety of the vertebral body, canal, pedicles, transverse processes, and spinous process as well as any paraspinal or epidural disease, if present. The clinical target volume (CTV) will include the involved index spine lesion(s) as well at least one spine level superior and inferior to the index lesion(s). Depending on site, treatment technique, and immobilization, the planning target volume (PTV) will consist of a 3–5-mm circumferential expansion only. Patients may be treated with 3D-CRT or IMRT at the discretion of the treating physician. The following fractionation schedules will be deemed equivalent and used in this study: 20 Gy in 5 fractions, 24 Gy in 6 fractions, and 30 Gy in 10 fractions [15].

### Radiofrequency ablation and vertebral augmentation

Patients randomized to RFA/PVA will undergo the procedure under moderate sedation in the angiographic suite or with monitored anesthesia care in the operating room. RFA/PVA will be performed with the patient in the prone position. Unipedicular or bipedicular approaches will be used depending on the anatomy and location of the metastatic spine disease. During the procedure, 10-G trocar needles will be placed via a transpedicular or parapedicular approach, and once positioned, RFA will be performed. All RFA procedures will be performed with the OsteoCool™ RF Ablation System (Medtronic Inc.) through the trocar needles. This consists of a coaxial, bipolar technology which delivers RF energy to the site of spine disease. The internally cooled ablation probes control the temperature which helps to keep RF heating within the desired treatment area and reduces the potential thermal damage to the adjacent tissue. Two probes may be used simultaneously, depending on the anatomy, to achieve larger ablation zones, and time adjustments can be made to control the size of the ablation zone. Once the RFA procedure has completed, PVA will be performed, as necessary, using 11-G needles advanced through the trocars and into the anterior 1/3 of the vertebral body. Kyphon high viscosity bone cement will be administered until the cement adequately fills the vertebral body/ablation zone or until the cement leakage precludes additional filling of the vertebral body. Kyphoplasty balloon augmentation will be performed at the discretion of the operator prior to cement administration. Once the entire procedure is completed, the patient will be transferred to the recovery area for a minimum of 3 h to allow the bone cement to solidify. Patients will be discharged the same or next day.

### Data management

MCI provides an in-house Clinical Scientific Review Committee (CSRC). The CSRC is responsible for reviewing all diagnostic, therapeutic, and non-therapeutic cancer research studies conducted at MCI. The MCI Data Safety and Monitoring Committee (DSMC) is responsible for assessing study progress, the reporting of adverse events and unanticipated problems, and the accuracy and integrity of the research data and protocol compliance. The DSMC is composed of representative physician experts and meets requirements as set forth from the NIH. This study will be reviewed by the MCI DSMC, independent from the investigator and study funder, every 3 months as per the assigned risk score. Both committees collaborate to ensure that the clinical trial is being conducted in regulatory compliance.

MCI studies are each assigned one or more Clinical Research Coordinators (CRCs) and Research Assistants (RAs) based on therapeutic area. CRCs are responsible to assist the PIs in the scheduling of patients and collection of study-related data from patients (surveys/questionnaires). RAs are responsible for data entry into MCI’s electronic data capture (EDC) system. MCI Data Management Coordinators (DMCs) are responsible for creating electronic Case Report Forms (eCRFs) for research study. RAs enter data into these eCRFs, which is then validated. At interim and/or final analysis, MCI DMCs export the study dataset and provide it to the PI and MCI Biostatistician for data analysis.

Data will be entered into MCI’s electronic data capture (EDC) system (21 CFR Part 11 compliant), which is a secured system used to collect and manage data for clinical research studies. This system is currently in use for MCI institutional studies. Patients will be identified by initials only (first, middle, last); if there is no middle initial, a hyphen will be used (first-last). Last names with apostrophes will be identified by the first letter of the last name. Data collected during the course of research will be kept strictly confidential and only accessed by members of the study team. Other staff employed by BHSF (sponsor) may have access to study participant data as part of their routine job duties. All staff are governed by the BHSF confidentiality policy and HIPAA guidelines.

### Statistical analysis

Based on the reported incidence of complete or partial pain response from the results of the feasibility matched cohort study of RFA/PVA and EBRT [8], with a two-sided *α* = 0.05 and *β* = 0.20 and a study design of 2:1 randomization scheme (RT plus PVA/RFA:RT), the study will be adequately powered with 52 patients (35 in the RT plus PVA/RFA arm and 17 in the RT arm). Assuming a 5% ineligibility rate, a death rate of 15%, and a patient non-compliance rate of 15%, the total sample size required would be 80 patients. No interim analyses with treatment efficacy results are planned. The primary endpoint is complete or partial pain response at 3 months after randomization, and all eligible, randomized patients will be included in the analysis regardless of treatment compliance (intent-to-treat analysis). Descriptive statistics of the actual change scores, mean change scores, and standard deviations will also be reported. For analysis of the secondary pain response endpoints (rapidity of pain response and duration of pain response), the median time to each outcome variable will be estimated using the Kaplan-Meier approach, the stratified log-rank test will be used to test for statistically significant differences with an α = 0.025, and the Cox proportional hazards regression model will be used to determine hazard ratios and 95% confidence intervals for the treatment differences. Moreover, unadjusted ratios and adjusted ratios for radiosensitive vs. radioresistant histology will be computed. The incidence of adverse events will be reported according to CTCAE v5.0, and differences in the rates at 3 months between the two treatment arms will be tested using the two-sided chi-square test at the 0.05 significance level. To analyze differences in QOL, global pain, and health utility, changes in FACT-G, BPI, and EQ-5D will be described with longitudinal data analysis. The mean differences will be compared across the treatment arms using the two sample *t* test at the 0.025 significance level. The relationship between treatment response and QOL will also be analyzed using the two sample *t* test at the 0.025 significance level.

### Ethics issues, information, and safety

The study protocol and patient informed consent documents were submitted to the Institutional Review Board at Baptist Health South Florida and approval was granted in 3/2020 (#1544454-2). As both treatment arms in this trial are currently approved in the routine care of patients with spine metastasis, insurance approval for the study treatment will be obtained from patients’ insurance providers. Interim reports with descriptive statistics will be prepared twice a year until the final study results are available. In general, the interim reports will contain information about the accrual rate with a projected completion date for the accrual phase, data quality, compliance rate of the treatment, and frequencies and severity of the adverse events. Unexpected adverse events (AEs) will be graded by the treating physician, assigned an attribution, and then reported to the principal investigator and study sponsor depending on grade and attribution using CTCAE v5.0. The interim reports will not contain treatment efficacy results with respect to the primary or secondary endpoints.

## Discussion

Spine metastasis is a common occurrence in patients with metastatic disease and results in pain, neurologic deficits, decline in performance status, disability, inferior quality of life, and reduction in ability to receive cancer-directed therapies. To date, there have been more than 20 randomized controlled trials, 32 prospective non-randomized studies, and 4 meta-analyses/pooled analyses regarding the role of palliative EBRT for patients with bone metastasis [15]. Despite this wealth of research, there are clear limitations to conventional EBRT, including modest rates of pain relief [3], high rates of disease recurrence [16], very low response rates for patients with various histologies (including sarcoma, melanoma, gastrointestinal, non-small cell lung cancer, and renal cell carcinoma) [17], and limited benefit in neurologic deficit improvement [17]. Therefore, the aim of this randomized clinical trial is to assess the efficacy of combined modality therapy with RFA/PVA together with EBRT compared to EBRT alone. We hypothesize that the addition of RFA/PVA may result in a robust and clinically meaningful improvement in the proportion of patients with spine metastasis experiencing pain relief at 3 months. Temporal analyses of the rapidity of pain response and duration of benefit will also provide important comparative outcomes. Finally, inclusion of key parameters of pain impact, health utility, and quality of life will lead to a better understanding of the relationship between metastatic disease of the spine and important patient-centric outcomes.

## Trial status

Version 3.0 of the protocol, revised last on April 7, 2020, received IRB approval on April 19, 2020. The study is currently open to patient accrual as of May 2020, and the accrual period is expected to last 18 months. Protocol amendments, if needed, will be reviewed by the study sponsor and submitted the IRB.

## Supplementary information

**Additional file 1.** SPIRIT 2013 Statement: Defined Standard Protocol Items.

## Data Availability

The principal investigator, co-investigator, and statistician for the protocol will have access to and be responsible for all of the data for this study.
